# Food security in a perfect storm: using the ecosystem services framework to increase understanding

**DOI:** 10.1098/rstb.2012.0288

**Published:** 2014-04-05

**Authors:** G. M. Poppy, S. Chiotha, F. Eigenbrod, C. A. Harvey, M. Honzák, M. D. Hudson, A. Jarvis, N. J. Madise, K. Schreckenberg, C. M. Shackleton, F. Villa, T. P. Dawson

**Affiliations:** 1 Centre for Biological Sciences, University of Southampton, Southampton SO17 1BJ, UK; 2 Centre for Environmental Sciences, Faculty of Engineering and the Environment, University of Southampton, Southampton SO17 1BJ, UK; 3 Social Statistics and Demography, University of Southampton, Southampton SO17 1BJ, UK; 4 LEAD, Chancellor College, University of Malawi, Zomba, Malawi; 5 Betty and Gordon Moore Center for Science and Oceans, Conservation International, 2011 Crystal Drive, Suite 500, Arlington, VA 22202, USA; 6 International Centre for Tropical Agriculture (CIAT) and CGIAR Research Program on Climate Change, Agriculture and Food Security (CCAFS), CALI AA6317, Colombia; 7 Department of Environmental Science, Rhodes University, Grahamstown, South Africa; 8 Basque Centre for Climate Change (BC3), IKERBASQUE, Basque Foundation for Science, Bilbao 48008, Spain; 9 School of the Environment, University of Dundee, Dundee DD1 4HN, UK

**Keywords:** agriculture–forest interface, ecosystem services, food security, Malawi, models

## Abstract

Achieving food security in a ‘perfect storm’ scenario is a grand challenge for society. Climate change and an expanding global population act in concert to make global food security even more complex and demanding. As achieving food security and the millennium development goal (MDG) to eradicate hunger influences the attainment of other MDGs, it is imperative that we offer solutions which are complementary and do not oppose one another. Sustainable intensification of agriculture has been proposed as a way to address hunger while also minimizing further environmental impact. However, the desire to raise productivity and yields has historically led to a degraded environment, reduced biodiversity and a reduction in ecosystem services (ES), with the greatest impacts affecting the poor. This paper proposes that the ES framework coupled with a policy response framework, for example Driver-Pressure-State-Impact-Response (DPSIR), can allow food security to be delivered alongside healthy ecosystems, which provide many other valuable services to humankind. Too often, agro-ecosystems have been considered as separate from other natural ecosystems and insufficient attention has been paid to the way in which services can flow to and from the agro-ecosystem to surrounding ecosystems. Highlighting recent research in a large multi-disciplinary project (ASSETS), we illustrate the ES approach to food security using a case study from the Zomba district of Malawi.

## 1. Introduction

In 2009, Sir John Beddington, the UK's chief scientist, described an oncoming ‘perfect storm’ scenario. Unless 50% more food, 50% more energy and 30% more freshwater were available by 2030, he argued, there would be simultaneous food, water and energy shortages on a global scale [1]. While there have been predictions for each of these key ‘commodities’ from 2050 onwards and in isolation, this ‘perfect storm’ metaphor has struck a chord with governments and civil society owing to the immediacy and complexity of the linked issues; pursuit of food security is inextricably linked to water and energy security, especially with the increasing demand and desire for biorenewables [2]. A key challenge highlighted in several recent high-level reports and reviews is how to increase food security sustainably in a climate-change-resilient manner, while reducing greenhouse gas emissions, alleviating poverty and conserving biodiversity [3–6].

It is important to recognize that food security is not just about increasing yields. Food security ‘exists when all people, at all times, have physical and economic access to sufficient, safe and nutritious food to meet their dietary needs and food preferences for an active and healthy life’ [7]. It is determined by four factors: (i) availability (from agricultural production and land-use or exchange); (ii) stability of supplies (e.g. seasonally and from year to year); (iii) access (dependent on financial means but also physical access and social factors); and (iv) biological utilization of food (e.g. nutritional diversity and food safety issues) [8]. Thus, increasing yields will address only one aspect of what makes individuals, households, communities and nations food secure or insecure. Addressing food insecurity requires multi-disciplinary perspectives and solutions.

Agricultural ecosystems are managed by humans largely to optimize provisioning ecosystem services (ES), such as food, fibre and fuel, yet these benefits depend upon regulating ES, for example pollination, from the wider landscape and environment for their long-term provision and sustainability [9]. Agriculture, in turn, also provides essential regulating, provisioning and cultural services to communities. Managing the cultivated agro-ecosystems and their interaction with uncultivated ‘natural’ ecosystems is thus not only important now, but will also become increasingly important while seeking to achieve or increase food security and maintain environmental integrity and resilience. Agriculture currently accounts directly for approximately 19–29% of global greenhouse gas emissions and is also the leading driver of deforestation and forest degradation globally, which accounts for an additional 17% of global carbon emissions [10]. For example, between 1980 and 2000, 83% of new croplands and pastures in the tropics were created at the expense of natural forests [11]. Reducing emissions from agriculture (e.g. through broad-scale adoption of ‘climate smart’ agriculture) and preventing the expansion of agriculture into remaining forested areas [12] must be central components of any mitigation plan. Indeed, ongoing policy discussions on REDD+ (Reducing Emissions from Deforestation and Forest Degradation and forest enhancement) recognize that tackling the drivers of deforestation and degradation, particularly agriculture, is the key for success [13]. The contribution of agriculture to biodiversity loss [14] means that finding ways to ensure that agriculture can meet the growing food demand without further degrading natural ecosystems is also critical for the overall goals of the Convention on Biological Diversity.

In its Ecosystem Management Policy report on Food and Ecological Security, the United Nations Environment Programme (UNEP) attempts to identify the trade-offs and synergies of these two objectives [15]. However, traditional approaches to investigating the interacting processes between food security and ES, e.g. based upon deterministic conceptualizations and neoclassical economics, fail to capture causality or even acknowledge that stocks of natural capital are dwindling and may already be too low to sustainably support long-term societal benefits, health and well-being. Power [16] is optimistic in suggesting that ‘there have been several recent advances in our ability to estimate the value of various ecosystem services related to agriculture, and to analyze the potential for minimizing tradeoffs and maximizing synergies’ (p. 2969). The Millennium Ecosystem Assessment and UNEP reports also offer optimism and solutions, while highlighting the need for future research to generate spatially and temporally explicit frameworks [15,17].

The ES framework—in which services and goods provided by natural and semi-natural ecosystems are explicitly linked to human well-being [18]—has considerable potential for managing land to achieve both food security and environmental sustainability. This is because both agricultural production, and services associated with agro-ecosystems, as well as more natural environments (i.e. the existence value of biodiversity, flood and climate regulation services), are encompassed by the framework.

Since the publication of the Millennium Ecosystem Assessment, the ES framework has become highly influential in both academia and policymaking. At international level, an example of this is the establishment in 2012 of the new Intergovernmental Platform for Biodiversity and Ecosystem Services. The Millennium Ecosystem Assessment has also been instrumental in generating numerous national ecosystem assessments [19–21] and ES valuation reports [22], resulting in the possibility of taking an ES approach to address global environmental challenges, such as in the World Bank's Wealth Accounting and Valuation of Ecosystem Services (http://www.wavespartnership.org/waves/) initiative, the International Council for Science and UNESCO's Programme for Ecosystem Change and Society initiative [23], and the UK's Ecosystem Services for Poverty Alleviation (ESPA, see http://www.espa.ac.uk/) research programme.

Surprisingly, in spite of this growing interest, few attempts have been made to explicitly outline how an ES framework can be operationalized to sustainably fulfil the multiple demands made of food security as well as addressing the need for environmental sustainability. Although the Convention on Biological Diversity has defined principles and guidelines for its ecosystem approach [24,25], the latter has a strong focus on the conservation and sustainable use of biological diversity. By contrast, the human well-being focus of the ES framework of the Millennium Ecosystem Assessment introduces additional challenges relating to the need to recognize and negotiate trade-offs between producers and beneficiaries of different services.

Here, we outline how the ES framework needs to be unpackaged to achieve food security in a sustainable manner. We argue that three key interlinked entry points are vital to achieving this: (i) understanding the spatial and temporal scale of ES; (ii) disaggregation of the beneficiaries of different ES and (iii) supporting the negotiation of trade-offs (including quantification, valuation and governance) between the demands of different ES users. We focus specifically on the application of such an approach for the rural poor at the forest–agricultural interface owing both to its importance for the 550 million people globally [26] who live in such areas and the linkages between natural and agro-ecosystems within them. We argue that the disaggregation of both beneficiaries and of the different elements of food security within the broader supply and use of ES, and quantification of both, are essential for effective management of ES for sustained human well-being and environmental sustainability in such regions.

We demonstrate this with an example of how an ES framework can be applied to research at the forest–agriculture interface in Malawi, where all the authors are engaged in a joint research project. One of the world's poorest countries, with a Human Development Index ranking of 170 out of 186 [27], Malawi had a population estimated at 13.1 million in 2008 growing at a rapid rate of 2.9% per year [28]. Some 39% of the population lives on less than US$1 per day [29]. The country's principal economic activity is agriculture, which is predominantly rain-fed, making it vulnerable to climatic shocks [30]. Deforestation rates range from 1 to 2.8% per year, varying across different parts of the country and across different types of forestry resources [28]. Malawi serves here as an example of many developing regions of the world where agricultural and forest products and services constitute the primary foundations of local livelihoods and well-being.

## 2. The relationship between food security and the environment

As highlighted by the food systems approach [31], the relationship between food security outcomes and the environment is complex and multi-directional. Thus, food security is not only dependent on (non-provisioning) ES but is also one of the greatest drivers of the loss of ES. The pursuit of food security through increased agricultural production, including through changes in land-use, land-cover, management practices and agricultural inputs (such as fertilizer, pesticides, irrigation), is a key driver of landscape change [15]. Even less-intensively managed types of provisioning services, for example harvesting of non-timber forest products (NTFPs), can lead to resource depletion, especially when harvested for commercial purposes, unless appropriate governance and education systems are in place [32,33].

**Figure 1.**
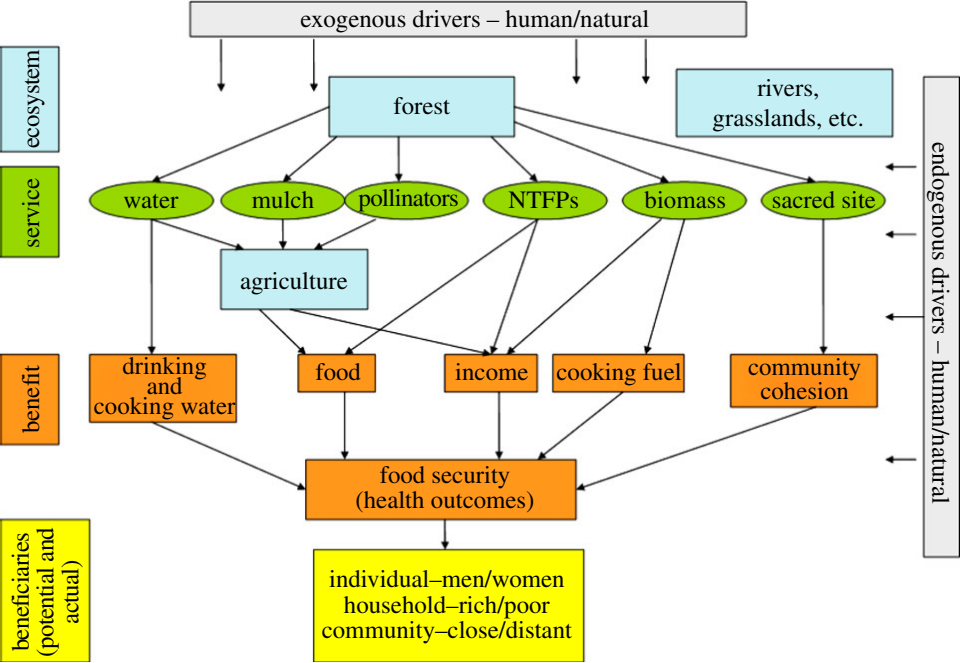
Schematic of the direct and indirect routes by which ES and benefits contribute to food and nutritional outcomes.

Food availability for many of the world's rural poor is particularly dependent on their being able to benefit from the flow of ES from non-agricultural ecosystems (figure 1). This may take many forms. The first is regular direct consumption of wild foods [34,35]. For example, wild foods account for over one-fifth of the diet of children in parts of South Africa and are particularly important for those from vulnerable households [36]. Secondly, wild foods are also often important safety nets for farmers when crops fail or food stocks run low [37,38]. Thirdly, food availability is further assured by ES to agriculture, from water for irrigation, timber for fencing and implements, to crop pollination [39–41] and pest regulation [22,42].

Food utilization is heavily dependent on the availability of fuelwood and clean water to enable households to prepare food safely [43]. Where people have poor access to fuelwood, they may be unable to cook or will shift to lower quality foods or eat fewer meals [44,45], all of which has impacts on health and nutrition, particularly of young children [46].

Incomes from trade in non-agricultural provisioning ES, such as fruit, nuts, fibres, resins and other NTFPs, also play a critical indirect role in food access by enabling households, particularly poor ones, to purchase food [47–49]. Less tangible contributions to food security from ecosystems include the use of sacred forests for harvest festivals [50] or, among the Loita Maasai, for initiation ceremonies conferring responsibility for different aspects of resource management [51]. These reserved sacred sites may also be important for the supply of other ES, such as pollination, pest control, fodder, biodiversity or water [52], which support a stable system of food security.

Flows of ES are shaped by complex and dynamic systems that operate over multiple temporal and spatial scales and often exhibit stochastic behaviour [53]. This complexity often makes it difficult to resolve an appropriate course of collective action to pursue sustainable livelihoods. In times of livelihood shocks (e.g. crop failure), the maintenance of flows of ES to the rural poor can become the only lifeline available [54]. In addition, the nature of many ES is dynamic, changing both in predictable cycles (e.g. seasonal) and in response to extreme events (such as floods, droughts, fires and pest outbreaks). Thus, the contribution of NTFPs to household livelihoods is known to be particularly important during the ‘lean’ or non-agricultural season [55,56]. Crises, for example Hurricane Mitch in Honduras, can bring to the fore the importance of forests as a ‘natural insurance’ for poor families [57]. The importance of direct use of ES is also a function of distances from the sites where specific ES are provided, such that the use declines with increasing opportunity costs of harvesting associated with increasing travel distances [58]. Thus, the loss of ES provisioning sites can severely disadvantage poorer households who subsequently have to travel much farther to obtain their needs. Within households, women may be the hardest hit; Sorenson *et al*. [59] highlight the disproportionate burden of water collection, which rests with women, particularly as the distance to the water source increases.

As indicated in figure 1, the relationship between food security and the environment can be affected by a range of internal and external drivers, of varying predictability and intensity. Communities may be able to adapt their use of ES in response to gradual trends, such as demographic changes and declining soil fertility. However, sudden shocks (e.g. droughts, earthquakes, commodity price collapses, disease or war), and unusual combinations of these in time or space, can lead to excessive pressure being exerted on ecosystems and cause degradation. The fact that developing countries typically have inadequate institutional ‘safety nets’, and inequitable access to fertile lands, resources and secure income often forces the rural poor to prioritize their short-term needs (i.e. feeding their families) over long-term sustainability [60]. Consequently, daily decisions for poor rural communities at the forest-agriculture interface are driven by coping strategies involving trade-offs of different ES. These decisions may be at odds with ES management priorities at other spatial or temporal scales.

## 3. Integration of food security and environmental sustainability within an ecosystem services framework

In this section, we examine in more detail the three elements of an ES framework we consider to be key in relation to achieving food security and environmental sustainability. There is a great deal of common ground between our first two elements (multiple scales of analysis and disaggregation of beneficiaries) and the five operational guidelines of the Convention on Biological Diversity [24]: however, our third element moves beyond what is provided for within the Convention's ecosystem approach by explicitly dealing with the trade-offs decision-makers need to make to achieve multiple outcomes (for example, delivering both food security and environmental sustainability) from one area.

### (a) Scales of analysis

As previously argued, the scale of analysis has a strong influence on how one views and describes a system. Focus on a single scale may obscure important processes at either finer or broader scales [60,61]. With respect to ES, it is necessary to consider not only biophysical processes but also institutional processes, which may operate at very different scales. Water catchments, for example, may cross several administrative boundaries, requiring the establishment of new forms of joint decision-making between upstream land managers and downstream beneficiaries. To achieve multiple goals (e.g. food production and ecosystem integrity and resilience), it is necessary to integrate across all pertinent spatial and temporal scales. Spatially, the local to national focus is important to really understand the local issues but these need to be set within a national context—i.e. identifying and understanding internal and external drivers of particular situations. At the forest–agriculture interface, which typically consists of a mosaic of more- and less-intensively managed habitats, there is a particular need for an integrated landscape approach to understand how the dynamic interactions between patches affect the delivery of ES [62–64].

Temporally, the flow of ES and human needs is not static [53]. Consequently, any analytical approach must embrace a wide range of temporal scales, fostering learning from past events and also giving a sense of the biophysical limits of what can be sustainably extracted from an ecosystem over time. Establishing institutional systems that can deal with the varying temporal scales (e.g. continuous, episodic, cyclical and stochastic) at which many biophysical processes operate is a particular challenge. This is further hampered by our lack of understanding of interconnectivity and feedback across overlapping scales within social–ecological systems [60].

### (b) Disaggregation of the beneficiaries

Recognition of the importance of disaggregating beneficiaries is relatively recent (e.g. [65]), but is now increasingly accepted to be critical for managing ES fairly [66]. We highlight the need to understand the disaggregation of two components. The first is to understand who benefits from different ES (in space and time) and how the benefits flow through to food production or other utility. This is a key difference to much policy-level food security work which deals with aggregated data for whole countries, regions or zones (e.g. [67]) as illustrated in the Food Estimation and Export for Diet and Malnutrition Evaluation (FEEDME) model (see box 1). The same applies to much ES work, where mapping scales are at catchment or larger scales, with limited explicit links to who the beneficiaries are for specific ES and where in the landscape they reside or farm [69]. Community and household-based studies are particularly important in disaggregating local beneficiaries (e.g. by gender or socioeconomic status) of different ES. However, for ES of global concern, such as biodiversity conservation and carbon sequestration, it is also important to determine how the needs of global beneficiaries interact with or affect the ability of local people to obtain the ES they need to support their livelihoods [70–72].

Box 1. **The food estimation and export for diet and malnutrition evaluation (FEEDME) model.**

**Figure 2.**
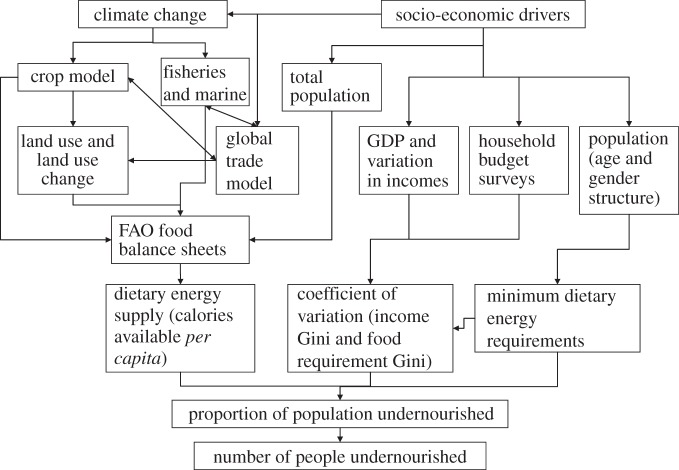
Schematic of the FEEDME model.

The FEEDME model has been developed for predicting climate change impacts on food security at a global scale (figure 2) and uses the Food and Agriculture Organisation (FAO) food energy deficiency methodology [68]. This methodology uses dietary energy supply, minimum dietary energy requirements and inequality in access to food to model the proportion of undernourishment within populations (at a national or local community scale).

**Figure 3.**
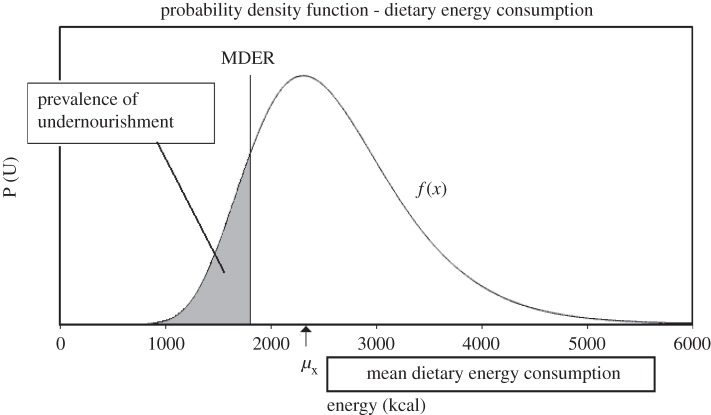
Distribution of dietary energy consumption.

— Food availability based on production, trade and non-food uses, etc. is recorded in a Food Balance Sheet (FBS), an account of all food items consumed at the household level, to calculate the mean dietary energy supply *μ*_x_ in calories *per capita* per day.

— Inequality in income and access to food is measured using a Gini coefficient, which is used to skew the distribution using a lognormal probability density function (figure 3).

— Minimum dietary energy requirement (MDER) per person per day is calculated using basic metabolic rate and physical activity levels according to the age and gender structure of the population. Population below this minimum cut-off is considered undernourished.

The FEEDME model uses projections of population and gross domestic product (GDP), world trade impacts, land use and agricultural productivity developed under future social-economic scenarios and associated climate change to estimate numbers of undernourished people in developing countries. Crop modelling data and a world trade model are used to update FBSs through changes in productivity and agricultural trade, respectively, as a percentage of change from a defined baseline. Changes in the amount of cropland, fisheries and livestock data can also be used to update FBSs.

The second dimension requiring disaggregation therefore pertains to governance: understanding who takes decisions about different ES (both about the management of the source ecosystem and the management of the flow of service, e.g. in irrigation or fuelwood-harvesting situations) and whether other users and stakeholders respect their authority to do so. Participatory engagement is the key to understanding this at the local level but this has to be combined with district- and national-level analysis to consolidate the official governance perspective on management activities with what is happening *de facto* on the ground. Increasingly, we must also consider the impacts of governance at the international level.

### (c) Consideration of trade-offs in policy- and decision-making

The two points above lead to the clear understanding that there are always going to be trade-offs between which services are prioritized from which ecosystems and for whom. Some authors go so far as to argue that all environmental management interventions in the Global South are likely to lead to both justices and injustices [73]. The greater the human demands on a landscape, and the less transparent or legitimate local governance or authority systems are, the more intractable the trade-offs (e.g. between provisioning, regulating and cultural ES) become [74,75]. We argue that taking an ES approach means firstly that there is a greater opportunity for integration, and hence a reduced need for trade-offs between social and ecological needs [76] and secondly, that trade-off decisions are made more transparently, and more equitable compromises can be reached that recognize the needs of current stakeholders and future generations in different locations. It is important to recognize, however, that the trade-off analysis is inevitably a risk-based process, especially where the lives of poor people are concerned. While priority should be given to those ES that can alleviate poverty and hunger in the short term, this should not lead to a reduced capacity in other critical ES on a sustainable basis. The challenge for policymakers is to adopt a risk evaluation method that can be used to analyse trade-offs and demonstrate causal relationships in future scenarios.

Achieving an integrated approach is supported by the widespread recent recognition in the ES literature of the importance of managing for ‘bundles’ of services [74] rather than individual services. Ecosystem ‘bundles’ are defined as sets of ES that repeatedly appear together across space or time [74]. Focusing on bundles rather than on individual services allows a way to consider the trade-off analysis in diverse landscapes shaped by both social and ecological forces and could be a powerful way of looking at agro-ecosystems [75,77]. This contrasts with many Payments for Ecosystem Services schemes which, by promoting maximization of a single marketed ES (like sequestered carbon or biodiversity), can reduce the flows of other services [78,79], thus constituting a potential risk to the achievement of food, energy or water security for certain beneficiaries. Achieving food security sustainably therefore requires examining bundle-based trade-offs between provisioning and other ES for multiple beneficiaries [66].

Although a variety of integrated platforms for spatial modelling of ES have been developed over the last few years [80–82], these have barely begun to enable the investigation of the interactions and complex trade-offs between services under different scenarios that are crucial to food security. Because ES are inherently process-based and connect complex systems whose dynamic nature is poorly understood, high levels of ingenuity are required to produce useful models without oversimplifying the system [83,84].

Novel methodologies, for example the ARtificial Intelligence for Ecosystem Services approach (ARIES: [81,82,85,86]), are designed to be applied in the data-scarce contexts that are typical of ES applications (even more so in developing countries) but without dismissing the complex and dynamic nature of the ES problem. Such methods are waiting to be put to the test in the highly complex, real-life contexts studied by projects, for example the ASSETS project (http://espa-assets.org/) in which all authors are involved. Delivering on the promise of addressing this complexity and its consequences, while remaining tractable and ‘scalable’ to different levels of detail and available information, remains a primary test case for the ability of twenty-first century science to address social needs and to usefully inform real-life decision-making workflows.

The results from advanced modelling frameworks, for example ARIES, must be linked to policy needs to use this information to design response mechanisms that are robust and consistent at multiple scales. The Driver–Pressure–State–Impact–Response (DPSIR) framework, which originated in social sciences over 30 years ago [87], is a useful analytical framework for developing conceptual understandings of such interactions and feedbacks for ES (e.g. [88]). On the surface, DPSIR appears to be limited by its linearity: *Drivers* (such as population increase) deliver *Pressures* (e.g. overharvesting of a natural resource), which change the *State* of the ecosystem or ES (e.g. the standing stock of NTFPs), which produces *Impacts* (e.g. in human well-being) which lead to *Responses* (policy change, switch to other resources, etc.). Rounsevell *et al.* [89] suggest that this perceived linearity obscures the feedbacks present in social–ecological systems, although others (e.g. [90]) point out that the DPSIR model explicitly accounts for feedbacks via responses (either by local agents or by policy on a wider scale) and can easily be adapted to deal with complex questions relating to the relationships between environmental change and ES and how society might adapt to maintain ES provision at the levels it needs to maintain or enhance human well-being. A key element of applying the DPSIR framework is that it must be used to define the right question, i.e. to deal with the local challenge of environmental degradation, societal need, agricultural policies, etc. For this reason, the questions should be framed by the relevant stakeholders via participatory approaches rather than imposed by external agencies or developed at a scale that may not be relevant.

Scenario-building exercises can also be immensely helpful to allow stakeholders to discuss different options [65]. While an inclusive process using the best possible data in formats accessible to the widest group of stakeholders can help to achieve a fairer outcome, political ecologists caution against the hopeful ideal of a simple evidence-based policy process [91]. Resolving trade-offs requires a process for valuing ES together with clear and transparent criteria for deciding whose values to prioritize. Methods for valuing ES are varied and remain contested [92]. This applies particularly to non-marketed ES, similar to some cultural and spiritual services, with the possible result that their real value to communities may be trivialized and not given proper weight in decision-making [93]. Thus scenario-building or DPSIR processes, at local or national level, must take account of power differentials and consider carefully who is recognized as eligible to take part in decision-making [94]. Most importantly, there should be transparency about the goals of the process, whether these are concerned primarily with environmental improvement or strive for poverty alleviation and perhaps even reduction of existing inequities [92,95].

## 4. Applying the ecosystem services framework to food security predictions: Malawi case study

Here, we use the example of Malawi to illustrate our proposed approach of managing ES for food and nutritional security considering each of the three elements outlined above in §3. Malawi is a country experiencing rapid population growth and significant land-use change already exacerbated by climate change. The country is characterized by persistent high fertility rates underpinning the high population growth. On average, Malawian women have more than five births, and as a consequence the population of the country has more than quadrupled in 50 years since 1960 to just under 15 million [96], placing an increasing strain on the country's natural resources [28]. By 2050, the UN predicts that Malawi's population will exceed 45 million. Although the uptake of family planning methods is increasing, there is still a preference for large families [97]. Approximately half of all children under the age of 5 years are chronically malnourished [97] and more than 50% of the population live below the national poverty line [98]. The Malawi Vulnerability Assessment Committee [99] estimates that the number of people at risk of being food insecure in October 2012 was 1.97 million (13%). In the following sections, we show how national-level estimates of undernourishment are important but may need to be grounded at local scale in order to design appropriate policy responses. In particular, we highlight the importance of disaggregating beneficiaries and of supporting a transparent negotiation of trade-offs among different ES users.

### (a) Linking national-scale predictions of undernourishment to the local level

At national level, the dietary energy provision-based methodology for rapid assessment of undernourishment has become the *de facto* standard indicator of food insecurity [100]. Taking advantage of national level annual FAO data on food production and dietary statistics [101], this approach has the advantages of being transparent and globally applicable using existing datasets. It can directly empower governments and development agencies to tailor policies that match national dietary needs with strategies and mechanisms that directly support those needs, such as agricultural production and international trade in food commodities. This methodology is based upon the premise that food deprivation is based on a comparison of usual household food consumption expressed in terms of dietary energy (kcal) with minimum energy requirement norms. Populations with food consumption below the minimum energy requirement are considered underfed [68]. Box 1 shows how the FAO methodology has been integrated into a conceptual framework with a range of climatic and demographic factors. The FEEDME framework can be used to develop future scenarios of climate, population and social-economic change.

**Figure 4.**
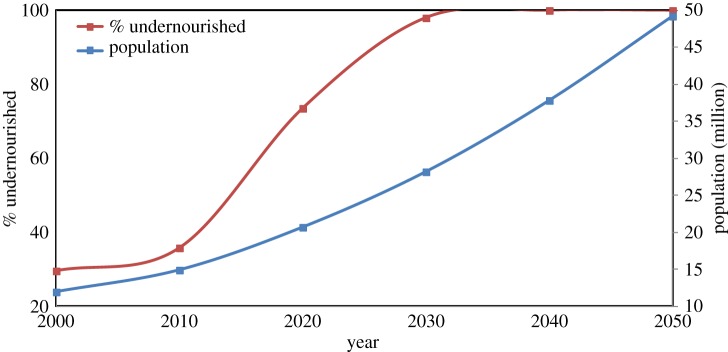
Population growth and % undernourishment projected from the FEEDME model for Malawi for the period 2000–2050 under the SRES A1b scenario (for assumptions and caveats, see text).

Taking Malawi as an example, we estimated current assessments and future changes in undernourishment using FEEDME for the period 2000–2050 using climate change and population drivers according to the Special Report on Emissions Scenarios (SRES) A1b scenario and the 2010 revisions of the UN medium variant population growth projections (figure 4). Although the SRES A1b, a high global emissions scenario leading to a mean global warming of around 2°C relative to the 1961–1990 baseline, is only one of many possible future greenhouse gas emissions trajectories, we used this, together with the UK Meteorological Office HadCM3 General Circulation Model, to highlight trends that support Beddington's ‘perfect storm’ scenario. Climate change impacts on food production were simulated by modifying food balance sheets through adopting results from crop simulation models [102]. The analysis presented here used changes in wheat and maize production as proxies for all C3- and C4- (photosynthetic pathway) type crops, respectively. While most crops (more than 85%) are classified as C3 types (wheat and rice being the two most important cereals on a global scale), C4-type crops (e.g. maize, millet, sugarcane and sorghum) are also very important food crops for many developing countries, including Malawi. The results (figure 4) show a dramatic increase in undernourishment which is clearly a function of both population growth and projected decline in primarily maize production which, on a global scale, shows a 35–40% mean reduction [103]. However, it should be noted that the model scenario assumed that the population demographics (age and sex structures), food inequality Gini coefficients, minimum dietary energy requirements and food imports all remained at the baseline values. These assumptions are important caveats, which mean that the projected proportion of undernourished people in figure 4 must be interpreted as an indicator of *potential exposure* to undernourishment. The steep increase from 2010 can therefore be read as an urgent need for some form of policy response. This might take the form, for example, of increasing yields through the adoption of improved technologies, clearing more land which decreases the supply of other ES, increasing food imports or reducing food exports, or promoting a different range of crops, or policies to slow down the population growth through voluntary schemes, for example meeting the demands for modern contraception. Approximately 26% of married women have an unmet need for family planning methods [97]. Currently, Malawi has adopted a Farm Input Subsidy programme to increase crop yield, though this is dependent on international donor support [104]. This scenario for Malawi is not unique. Many developing countries with growing populations face enormous challenges to meet their future food needs. This will require international efforts as well as national- and local-level changes in land use to increase food production and sustainable population growth.

While the FAO methodology and FEEDME projections for current and future undernourishment assessments are useful for identifying potential trends and responses at the national and policy level to strengthen food security, the approach is not very helpful at the community and landscape scales, or in recognizing the links between food production, food security and ES which underpin this production. This is because the national-level food balance sheets used in the FAO methodology are generated from aggregated agricultural statistics. These are frequently unreliable as illustrated in the case of Malawi by the discrepancy in maize yields for 2006/2007 reported by the Ministry of Agriculture and the National Statistics Office [104]. Although yields per hectare were similar in the two reports, the Ministry of Agriculture assumed the number of rural households was 1 million higher than the National Statistics Office, with the inflated number of households thought to be linked to the popularity of the Farm Input Subsidy programme [104]. The implications are not trivial as the inflated number of households results in national production estimates that suggest the country is producing more than 4000 calories per person per day, which is more than double the amount commonly assumed [104].

Another problem with agricultural statistics is that they do not account for foodstuffs that are not traded commercially, or more traditional crops or wild foods within the agro-ecosystems and fields. For example, in South Africa, High & Shackleton [105] reported that over 30% of the value of all edible plants harvested from homestead plots and gardens came from non-conventional or wild species. A good example of this in Zomba District, Malawi, is a vegetable dish made from wild-harvested orchid tubers, locally known as Chikande.

In addition, for the very poorest and rural communities, their diets are likely to be significantly different from the national food consumption lists thereby making the relationship between access to food (based upon income) and dietary intake uncertain. For these reasons, it is necessary to identify ways to bridge the gap in food security from the national to local levels, and ensure different spatial scales of analysis are integrated. This requires information and insights at the local scales, which can be gained through a variety of approaches, such as food diaries, focus group discussions, participant/household observation and the use of participatory rural appraisal techniques.

### (b) Disaggregating ecosystem service beneficiaries in terms of their food security needs

A growing body of literature highlights the particular importance of wild foods to children, HIV/AIDS-affected households and the poor [36–38,106]. This pattern is evidenced in Malawi. For example, dependence on forest reserves and customary forests for fuelwood is the greatest among poorer households [107]. Kamanga *et al*. [48] reported that in Chiradzulu district, the poorest households obtained 65% of their cash and non-cash income from NTFPs, compared with 25% from agricultural production and that the relative proportion declined with increasing wealth. Abu-Basutu [108] found at two sites in South Africa that HIV/AIDS vulnerable households had lower calorie intakes than non-vulnerable households, despite the higher need, and that in both sites males in the age groups aged 14–30 in vulnerable households had daily calorie intakes significantly below those recommend by the FAO. Females and all other age groups of males had satisfactory calorie intakes. The costs of obtaining essential ES may fall on different people within a household, as is the case for water collection in Malawi, which is predominantly considered a job for girls and women. Despite this recognition, the majority of studies on ES assume, implicitly or otherwise, that a given ES has a single value for all beneficiaries (see [109] for a rare large-scale study that considers the ability of beneficiaries to replace ES in their mapping of services).

We suggest that there are several complementary approaches to identify the relative contributions of ES to food security for different groups of beneficiaries. One of these is to use participatory rural appraisal methods [110,111] to prioritize, and hence value, the most important ES (in relation to food security) for different social groups. Such prioritization should use local criteria (as opposed to opportunity cost which is the dominant economic approach to value communities’ use of provisioning ES), such as taste, frequency of use, volume of use, availability during times of stress, accessibility or cultural requirements (e.g. [112]). In Malawi, participatory methods have served to highlight the importance not just of wild foods but also of income obtained from ES (such as through sale of fuelwood and charcoal) as coping strategies during periods of food insecurity [113,114]. Such participatory datasets can then be linked to spatially explicit maps showing the source of the prioritized ES in the landscape (e.g. [115]). By combining these maps with models of the impacts of climate change on ES, e.g. through the use of FEEDME for crop yields or KINEROS (kinematic runoff and erosion model) combined with GIS AGWA (Geographic Information Systems Automated Geospatial Watershed Assessment) for water flow projections [116], scenarios can be developed of current and future food and nutrition security faced by local populations.

While models similar to FEEDME can project estimates of per-capita energy (calories) provision as an important aspect of food security, a more complete picture of the role ES play for food security might focus on obtaining data on macronutrients or protein intake as an important part of peoples’ diets or dietary diversity. A good example of this approach is demonstrated by Golden *et al*. [106] who showed that removing access to bushmeat for a forest community in Madagascar would increase anaemia in children by 29% and triple the number of cases in the poorest households. Work by Kaschula [117] showed that HIV/AIDS vulnerable households had lower dietary diversity, unless they had access to wild foods which could compensate for the loss of certain purchased foods (owing to declining household income from increased healthcare demands and perhaps the loss of income owing to ill health) or self-produced crops owing to labour shortages. To gain this level of understanding of nutritional outcomes participatory methods are required, such as food diaries (e.g. [36]), health diaries and mapping [118], and anthropometric measurements (e.g. [119]). The latter typically involve measuring the height, weight and mid-upper arm circumference of children under the age of five since nutritional deficiencies are most notable among this age-group, and often have devastating and long-lasting consequences [120,121].

Large-scale datasets which contain information on the nutritional status of young children (and their mothers) exist (for example the Malawi Demographic and Health Surveys (MDHS) conducted every 5 years and the Malawi Integrated Household Survey (MIHS) also collected on a 5-yearly basis). Both the MDHS and MIHS collect some data on food consumption. In the case of the former, the focus is on children under the age of 5 years and on food consumed in the 24 h before the survey. The MIHS collects data on household food consumption but this is not accurately measured, and thus the calorific estimates are crude. Additionally, since these surveys are not repeated at household level, it is not possible to assess the seasonal changes in dietary intake or the nutritional status of individuals. By conducting repeated household surveys and anthropometric measurements in different seasons and improving on the estimation of calorific inputs using dietary diaries and food scales, it is possible to begin to understand the transitions into food insecurity and poor health of the rural poor and how much they depend on ES for their well-being (e.g. [106]).

### (c) Recognizing trade-offs between ecosystem services for food security

Understanding the direct and indirect contributions of ES to food security of different social groups is necessary to recognize and manage the trade-offs that may occur. This extends to the consideration of the clear temporal dimensions (both in terms of seasonal support and in times of stress) of dependence on ES [36,37,122,123]. A typical example is the commercialization of NTFPs by elites, which can lead to the loss of the resource as a safety-net for the poorest [32,33]. In Malawi, this applies to the trade in firewood or charcoal [48], which may undermine supplies at a local level for direct household needs and, if not managed sustainably, may lead to the degradation of other provisioning and regulating ES. Here, we hypothesize about how the use of a combination of a DPSIR process and a modelling platform, such as ARIES, could help to improve the outcome of ES trade-offs in relation to food security and environmental sustainability.

The charcoal trade employs over 200 000 people [124] and is considered to be the third largest industry in the country (valued at over US$40 million or 0.5% of GDP), yet produces no official revenue [125]. In spite of legal production being possible there was no single case of legal charcoal production in 2010 [125]. Charcoal is predominantly (but not exclusively) produced by men [126]. Although charcoal is considered to be a key contributor to deforestation [28], as yet there appears to be no information about how this ES may conflict with the use of the same resource for firewood, which is predominantly collected by women and children, and for other ES that play an important role for food security such as NTFPs, bushmeat, drinking and irrigation water. The conflict between these ES uses can only get worse as the charcoal market is expected to double by 2023 [124].

Participatory mapping of the source of different ES at local level and quantification of their use by different societal groups are the first step towards achieving more equitable management. As some of the drivers of the charcoal trade (e.g. urban fuel demand) are external to the local level, it is imperative that discussions are held at multiple levels. Stakeholder workshops bringing together community representatives with district and national decision-makers are needed. In line with the call of the Convention on Biological Diversity for intersectoral collaboration, such workshops can only be useful if they bring together people with an interest in food security as well as those concerned with energy and the environment. Informed decision-making can be supported through the use of modelling platforms, such as ARIES, to present information about flows of different ES to different beneficiaries as well as playing through different policy scenarios. While one option might be to further restrict access to forest resources, this has been found to lead to increased income inequality [48,127].

Experience from elsewhere [128,129] suggests that greater decision-making at local level might lead to a possible win–win scenario in which community-based natural resource management (CBNRM) plans ensure sustainable charcoal production. Participatory work on understanding natural resource governance at local level might explain why options for CBNRM and legal charcoal production—which are already available in the national policy—have not been taken up in practice and lead to modifications in their implementation. In the area around Zomba, understanding the impacts of different forest use scenarios on ES, such as water flow and siltation, could open the door for the local hydroelectric power producer to support CBNRM in communities to ensure more regular electricity supplies (M. Longwe 2012, personal communication). Understanding which beneficiaries benefit from which ‘bundles’ of ES could be important to determine how costs and benefits of land-use changes are allocated. Similar scenario building based on a disaggregated understanding of ES flows and beneficiaries could be helpful for decisions on proposals for large-scale land-use conversion to bioenergy crops. For example, a *Jatropha* oil-processing plant has become operational in Lilongwe and there is pressure on Mkuwazi forest in Northern Malawi for conversion to sugarcane production.

These examples highlight that, with explicit understanding of disaggregated sources and beneficiaries of ES, and the complex relationships between ES and food security at different scales, appropriate response mechanisms can be designed through policy and education. The translation of those diverse drivers and pressures into tangible and quantifiable directions for policymakers is vital and requires the development and implementation of new strategies that support the needs of local communities. While changes are taking place on a global scale, the effects at local and regional levels are poorly understood, hard to predict and thus difficult to mitigate with appropriate policy actions. Sahley *et al*. [30] argue that the debate about how best to achieve food security in Malawi has been concentrated too much at the national level between government and donors, rather than engaging civil society and the private sector. The FEEDME results highlight the dangers of policies made to address issues observed at the national scale having potentially dire consequences at the local level when ‘hidden’ ES benefits are not explicitly understood or taken into account. For example, many poor people survived the Malawi famine in 2001/2002, which was primarily caused by failed maize crop production through flooding and drought, by harvesting food, such as fruits and bush millet from remaining natural habitats. It is easily conceivable that the national response to challenges highlighted with the FEEDME analysis presented earlier may result in increased pressure on natural habitats through more natural land conversion to cropland. This may in turn generate perverse incentives for local communities, which increase pressure on the ES that they depend on. Conversely, the application of the ES framework, as we have outlined above, could lead to the recognition of the spatially and temporally differentiated importance of ES to different societal groups. A clear goal to resolve trade-offs to the benefit of the poorest could give rise to a response which focuses on improving the enabling environment (e.g. provision of community natural resource management rights and expertise) needed to support the coping strategies of the rural poor, ultimately contributing to greater food security and more sustainable use of the environment.

## 5. Conclusion

Many forecasts of the future show that human development cannot continue on the current trajectory without large-scale changes in ES that underpin human well-being. Using the example of the ES framework as applied to promoting or ensuring food security, we have demonstrated the inextricable links between ES and human well-being, illustrated by the situation in Malawi as a typical developing country. From this, we offer the following conclusions:

(i) Business as usual in science, agriculture and ecosystem management is not going to be sufficient to meet the challenges of near- and longer term futures, especially in the face of ‘perfect storm’ combinations of stressors. We need novelty in the questions asked and the frameworks used.
(ii) Food security represents the epitome of needing the natural and social sciences to work together in interdisciplinary ways and, for implementation, in transdisciplinary ways. The ES framework is a potentially useful vehicle for this. Agriculture and food security are moulded by (non-agricultural) ES and ES are impacted by agriculture, population increase and climate change, making the relationship both complex and very challenging.
(iii) Because of the need for novelty and inter- and transdisciplinary cooperation, bundling of conceptual and analytical frameworks is the key. No single framework can capture all salient aspects of the system at appropriate resolutions and at multiple scales. If used at multiple scales, and with a view to impacts on different societal groups, the DPSIR framework may be helpful by explicitly focusing on drivers of different kinds, their impacts and possible responses.
(iv) Average outcomes, predictions and policies are insufficient as demonstrated in our FEEDME example from Malawi. There must be appropriate resolution to identify who are the vulnerable, when, where and why, and how these change with context and time. Uncertainty is a reality and needs to be integral to frameworks used.

Agro-ecosystems, including managed forests and pastures, cover almost 40% of the Earth's terrestrial surface [130]. As major providers and consumers of ES, effective and sustainable management of these agro-ecosystems is essential within the broader landscape. We conclude that implementing the ES framework to understand and negotiate trade-offs at the critical interface between agro-ecosystems and less transformed systems is a promising way forward for meeting the simultaneous needs of food security and environmental sustainability.
